# Clinical Trials of Broadly Neutralizing Monoclonal Antibodies for Human Immunodeficiency Virus Prevention: A Review

**DOI:** 10.1093/infdis/jiaa377

**Published:** 2020-07-01

**Authors:** Sharana Mahomed, Nigel Garrett, Cheryl Baxter, Quarraisha Abdool Karim, Salim S Abdool Karim

**Affiliations:** 1 CAPRISA, Centre for the AIDS Programme of Research in South Africa, Durban, South Africa; 2 Department of Public Health Medicine, School of Nursing and Public Health, University of KwaZulu-Natal, Durban, South Africa; 3 Department of Epidemiology, Mailman School of Public Health, Columba University, New York, New York, USA

**Keywords:** monoclonal antibodies, broadly neutralizing antibodies, HIV, clinical trials

## Abstract

Passive immunization with broadly neutralizing antibodies (bnAbs) is a promising approach to reduce the 1.7 million annual human immunodeficiency virus (HIV) infections globally. Early studies on bnAbs showed safety in humans, but short elimination half-lives and low potency and breadth. Since 2010, several new highly potent bnAbs have been assessed in clinical trials alone or in combination for HIV prevention. Published data indicate that these bnAbs are safe and have a half-life ranging from 15 to 71 days. Only intravenous VRC01 has advanced to an efficacy trial, with results expected in late 2020. If bnAbs are shown to be effective in preventing HIV infection, they could fast-track vaccine development as correlates of protection, and contribute as passive immunization to achieving the goal of epidemic control. The purpose of the current review is to describe the current status and provide a synopsis of the available data on bnAbs in clinical trials for HIV prevention.

## Teaser

The use of broadly neutralizing antibodies (bnAbs) is a promising approach that could help reduce the human immunodeficiency virus (HIV) epidemic burden. This review describes the status and available data on bnAbs in clinical trials for HIV prevention.

Human immunodeficiency virus (HIV) remains a major public health concern, with 37.9 million people living with HIV and 1.7 million new infections globally in 2018 [1]. In the absence of an effective vaccine, new strategies for HIV prevention are a priority, especially for vulnerable populations in high HIV burden settings. The use of broadly neutralizing antibodies (bnAbs) is a promising approach that could contribute to a reduction in the epidemic burden, until an effective vaccine is available.

From the late 1980s, the use of inactivated hyperimmune plasma from HIV positive donors was evaluated for suppression of viral replication. Studies demonstrated that the administration of antibodies to the HIV core antigen (anti-p24) resulted in clearance of p24 antigen in the blood for up to a period of 11 weeks. Subsequent studies showed that the administration of HIV-specific antibodies could lead to a clinically significant decline in plasma virus [2].

Antibody-based therapy for prevention is decades old [2]. Serum therapy, the transfer of blood serum containing immunoglobulins to treat diseases such as diphtheria and tetanus, has been available since the early 1900s [3]. Immunoglobulins were subsequently purified and used for both preexposure and postexposure prophylaxis in infectious diseases. The concept of passive immunization has been utilized in diseases such as diphtheria, tetanus, hepatitis B, and botulism; however, a licensed immunoglobulin is only available for anthrax, respiratory syncytial virus in premature infants and for rabies [3, 4].

**Figure 1.**
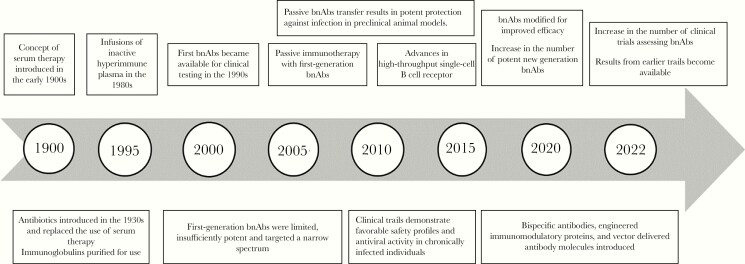
Milestones of antibody development for clinical use. Abbreviation: bnAbs, broadly neutralizing antibodies.

**Figure 2.**
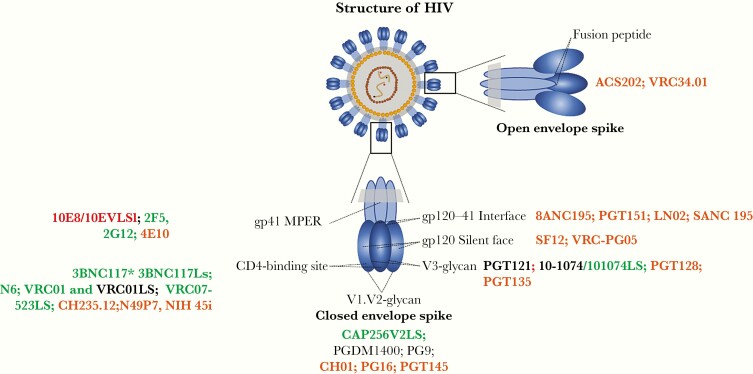
Human immunodeficiency virus (HIV)–specific neutralizing antibody targets with broadly neutralizing antibody (bnAb) candidates in ongoing (*green*), completed (*black*), and paused (*red*) clinical trials. Candidate bnAbs in clinical development (*orange*) are included here for completeness but are not included in the review. Abbreviations: gp, glycoprotein; MPER, membrane-proximal external region.

In the field of HIV, the antibody development pipeline has advanced rapidly (Figure 1). The first bnAbs became available for clinical testing in the 1990s [2]. Through technological advances, more potent bnAbs were discovered from 2010 onward [5]. The current bnAbs assessed in trials, target conserved neutralization-sensitive epitopes on the HIV-1 envelope (Figure 2). The mechanisms of action of these bnAbs include the disruption of the glycan shield of the HIV envelope trimer in 5 regions: the V2 apex, the V3 glycan site, the CD4-binding site, the fusion peptide, which includes the site for cleavage of glycoprotein (gp) 160 into gp120 and gp41, and the membrane-proximal external region (MPER). Recently, a sixth site was discovered, defined by the bnAb VRC-PG05, which binds to the center of the so called “silent face” of gp120 [6].

In addition to neutralization, bnAbs have a myriad of nonneutralizing antibody functions. The antigen-binding fragment (Fab) portion of the antibody interacts specifically with the cognate part of the protein that it recognizes. The fragment crystallizable region (Fc) portion of the antibody has the ability to interact with the Fc receptors on effector cells, such as natural killer cells or phagocytes for antibody-dependent cellular cytotoxicity and antibody-dependent cellular phagocytosis, respectively [5].

Several preclinical studies have shown that bnAbs administered passively are able to protect animals from simian-human immunodeficiency virus (SHIV) infection and treat nonhuman primates (NHPs) [5, 6]. Several trials are currently underway, and data from these trials will contribute to advancing the antibody field. If bnAbs are to be routinely used, additional factors must be considered, such as cost-effectiveness, ease of administration, and mass production [7]. The purpose of the current review is to describe the status of bnAb trials for HIV prevention and provide a synopsis of the available data.

## METHODS

All trials were identified through the search portal of the World Health Organization’s International Clinical Trials Registry Platform. The Web site was last accessed on 31 May 2020. This search focused on antibodies that have advanced the clinical development pipeline. A search string for each bnAb was applied for: VRC01, VRC07-523LS, CAP256V2LS, PGT121, PGDM1400, N6, 10E8,10EVLS, PG9, 3BNC117, 10-1074, and 2G12. Only trials that evaluated healthy, HIV-uninfected individuals with the aim of HIV prevention were reviewed. Completed trials with published data, for the period from 2010 to date, were then searched for in PubMed (Figure 2).

## RESULTS

**Figure 3.**
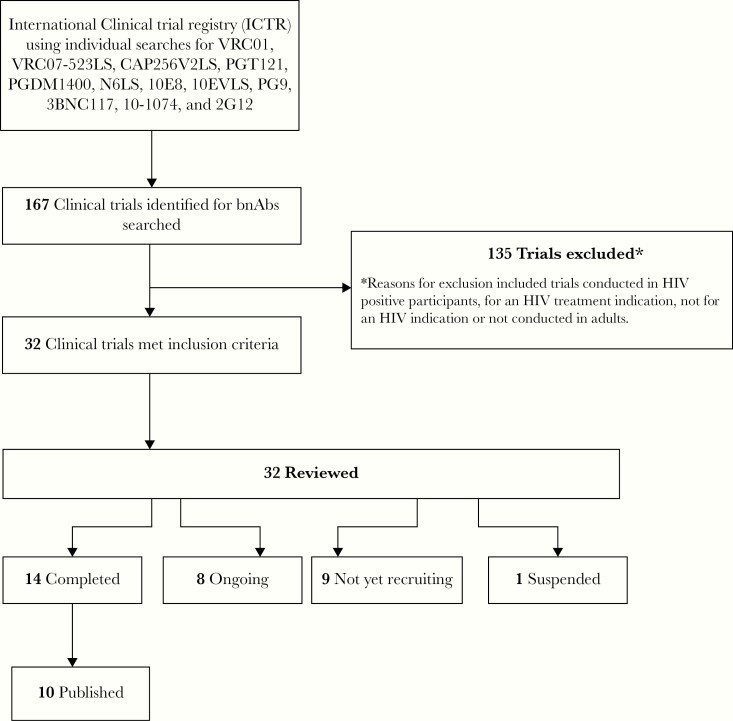
Search methods and results. Abbreviations: bnAbs, broadly neutralizing antibodies; HIV, human immunodeficiency virus; ICTRP, International Clinical Trials Registry Platform.

A total of 167 trials registered on the International Clinical Trials Registry Platform were identified. Of these, 135 trials did not meet the eligibility search criteria (Figure 3). These were bnAb trials for HIV treatment rather than prevention, trials not designed for an HIV indication, or trials not conducted in adults. A total of 32 trials met the inclusion criteria. Fourteen of these trials were completed, and 1 was suspended owing to safety concerns. Of the 14 completed trials, 10 trials with published data were included in this review. For completion, data from 2 conference abstracts were also included.

### Early bnAbs in Trials

Early studies evaluated 2G12, directed against gp120, alone or in combination with 2F5 and 4E10, both directed against the MPER region. These studies demonstrated safety and reduction in HIV RNA levels in plasma of HIV-positive individuals [8, 9]. In addition, trials describing F105, hNM01, and KD-247 demonstrated limited potency and breadth. Overall, these bnAbs were safe, but most had short elimination half-lives, ranging from 3 to 13 days, with 2G12 ranging from 14 to 22 days, and lacked potency and breadth [2].

In the early 2000s, 2F5, 2G12, and 4E10 were assessed as combination topical agents. The MABGEL trial was the first trial that evaluated bnAbs in the female genital tract and demonstrated safety with favorable pharmacokinetics in vaginal secretions [10]. Median antibody levels were 7.74, 5.28, and 7.48 mg/mL, respectively, for 2F5, 4E10, and 2G12, 1 hour after high-dose MABGEL administration, but the levels declined rapidly thereafter, with estimated half-lives of 1–4 hours. No evidence of systemic absorption was observed. The combination of 2F5, 2G12, and 4E10 was also administered intravenously to virologically suppressed participants on antiretroviral therapy (ART) and was able to delay viral rebound. Escape mutant analysis showed that 2G12 was essential for the in vivo effect [11].

In the Pharma-Planta project, 2G12 was expressed in transgenic tobacco and became the first antibody product derived from plant biotechnology that was approved by pharmaceutical regulators for clinical testing [12]. The HIV-neutralizing median inhibitory concentration for the clinical batch was 21 μg/mL, and after vaginal administration at varying dosing levels, 2G12 was well tolerated, safe, and not systemically absorbed. Although vaginal fluid samples were compromised during transportation, analysis revealed that 2G12 was still detectable in samples taken up to 8 hours after administration. Further studies following this trial demonstrated that plants, in particular *Nicotiana benthamiana*, were suitable to produce bnAbs, highlighting a cheaper and potentially scalable method, with particular implications for low-income settings [13].

Although all the earlier bnAbs were generally safe in phase 1 trials, only 2G12 advanced through the clinical development stage. This was owing to the advent of more potent bnAbs with broader coverage and longer half-lives.

### Current bnAb Trials

Since 2010, several potent bnAbs have entered trials(Figure 2).

### 3BNC117 and 10-1074

3BNC117 and 10-1074, were found to be safe when administered intravenously alone and in combination to HIV-uninfected individuals [14, 15]. The half-lives of 3BNC117 and 10-1074 were 17.2 and 24 days, respectively, and both antibodies maintained neutralizing activity in serum [14, 16]. When administered in combination, the half-lives of both antibodies did not change significantly [17]. These bnAbs are now developed for subcutaneous administration for HIV prevention, and the lysine-serine mutation (LS) versions of both bnAbs, with extended serum half-lives, are currently in phase 1 trials (Table 1).

**Table 1.** Clinical Trials Investigating Broadly Neutralizing Antibodies for Prevention of Human Immunodeficiency Virus

Study Product	Combination Arms	Clinical Trial Identifier	Scientific Description	Trial Status	Publication
VRC01	NA	NCT01993706	VRC 602: Phase 1 dose-escalation study of the safety and pharmacokinetics of a human monoclonal antibody, VRC-HIV MAB060-00-AB(VRC01), administered intravenously or subcutaneously to healthy adults	Completed	Ledgerwood et al (2015) [19]
VRC01	NA	NCT02165267	Phase 1 clinical trial to evaluate the safety and drug levels of a human monoclonal antibody, VRC-HIVMAB060-00-AB (VRC01) administered in multiple doses intravenously and subcutaneously in different dosing schedules to healthy, HIV-uninfected adults	Completed	Mayer et al (2017) [20]
VRC01; VRC01LS	NA	NCT02599896	VRC 606: Phase 1, dose-escalation study of the safety and pharmacokinetics of a human monoclonal antibody, VRC-HIVMAB080-00-AB (VRC01LS) and VRC-HIVMAB060-00-AB (VRC01), administered intravenously or subcutaneously to healthy adults	Completed	Gaudinski et al (2018) [21]
VRC01	VRC01 + HSV8-N	NCT02579083	Phase 1, single-center study to assess the safety of MB66, a combined anti-HIV (VRC01-N) and anti-HSV (HSV8-N) monoclonal antibody film for vaginal application as microbicide	Recruiting	…
VRC01	NA	NCT02716675	Phase 2b study to evaluate the safety and efficacy of VRC01 broadly neutralizing monoclonal antibody in reducing acquisition of HIV-1 infection among men and transgender persons who have sex with men	Completed	…
VRC01	NA	NCT02568215	Phase 2b study to evaluate the safety and efficacy of VRC01 broadly neutralizing monoclonal antibody in reducing acquisition of HIV-1 infection in women in sub-Saharan Africa	Active, not recruiting	…
VRC01	NA	PER-019-16	Phase 2b study to evaluate the safety and efficacy of VRC01 broadly neutralizing monoclonal antibody in reducing acquisition of HIV-1 infection among men and transgender persons who have sex with men	Completed	…
VRC01; VRC01LS	NA	NCT02797171	Phase 1 clinical trial to evaluate the safety, pharmacokinetics, and antiviral activity of VRC-HIVMAB060-00-AB (VRC01) and VRC-HIVMAB080-00-AB (VRC01LS) in the serum and mucosa of healthy, HIV-uninfected adult participants	Completed	…
VRC01	NA	NCT03729752	PET imaging of radiolabeled anti–HIV-1 envelope monoclonal antibody (VRC01)	Recruiting	…
VRC07-523LS	NA	NCT03015181	VRC 605: Phase 1 dose-escalation study of the safety and pharmacokinetics of a human monoclonal antibody, VRC-HIVMAB075-00-AB (VRC07-523LS), administered intravenously or subcutaneously to healthy adults	Completed	Gaudinski et al (2019) [22]
VRC07-523LS	VRC07-523LS + 10E8VLS	NCT03565315	VRC 610: Phase I safety and pharmacokinetics study to evaluate a human monoclonal antibody (MAB) VRC-HIVMAB095-00-AB (10E8VLS) administered alone or concurrently with MAB VRC- HIVMAB075-00-AB (VRC07-523LS) via subcutaneous injection in healthy adults	Suspended	…
VRC07-523LS	VRC07-523LS + PGT121	PACTR201808919297244	Phase 1 study to determine the safety and pharmacokinetics of the human monoclonal antibodies, VRC07-523LS and PGT121 administered subcutaneously to HIV-negative adults in South Africa	Enrolment completed: follow-up	…
VRC07-523LS	PGT121 + VRC07-523LS; PGT121 + VRC07-523LS + PGDM1400	NCT03721510	Phase 1/2a study of PGT121, VRC07-523LS and PGDM1400 monoclonal antibodies in HIV-uninfected and HIV-infected adults	Recruiting	…
VRC07-523LS	PGT121 + VRC07-523LS; PGDM1400 + VRC07-523LS; 10-1074 + VRC07-523LS; PGDM1400 + PGT121 + VRC07-523LS	NCT03928821	Phase 1 clinical trial to evaluate the safety, tolerability, pharmacokinetics, and antiviral activity of combinations of monoclonal antibodies PGT121, PGDM1400, 10-1074, and VRC07-523LS administered via intravenous infusion in healthy, HIV-uninfected adult participants	Active, not recruiting	…
VRC07-523LS	NA	NCT03735849	Phase 1 clinical trial to evaluate the safety and pharmacokinetics of VRC-HIVMAB075-00-AB (VRC07-523LS) in the sera and mucosae of healthy, HIV-1–uninfected adult participants	Active, not recruiting	…
VRC07-523LS	NA	NCT03387150	Multicenter, randomized, partially blinded phase 1 clinical trial to evaluate the safety and serum concentrations of a human monoclonal antibody, VRC-HIVMAB075-00-AB (VRC07-523LS), administered in multiple doses and routes to healthy, HIV-uninfected adults	Active, not recruiting	…
VRC07-523LS	PGDM1400 + PGT121 + VRC07-523L; PGDM1400 + PGT121	NCT03205917	Phase 1 randomized placebo-controlled clinical trial of the safety, pharmacokinetics and antiviral activity of PGDM1400 and PGT121 and VRC07-523LS monoclonal antibodies in HIV-uninfected and HIV-infected adults	Active, not recruiting	…
VRC07-523LS	VRC07-523LS + PGT121.414.LS	NCT04212091	Phase 1 dose-escalation clinical trial to evaluate the safety, tolerability, pharmacokinetics, and antiviral activity of the monoclonal antibody PGT121.414.LS administered alone and in combination with VRC07-523LS via intravenous or subcutaneous infusions in healthy, HIV-uninfected adult participants	Not yet recruiting	…
CAP256V2LS	CAP256V2LS + VRC07-523LS CAP256V2LS + PGT121	PACTR202003767867253	Phase I dose-escalation study of the safety, tolerability and pharmacokinetics of a human monoclonal antibody, CAP256V2LS (VRC-HIVMAB0102-00-AB) administered intravenously to HIV-negative and HIV-positive women or subcutaneously alone and in combination with VRC07-523LS and /or PGT121 to HIV-negative women in South Africa	Not yet recruiting	…
PGT121	NA	NCT02960581	Phase 1 randomized placebo-controlled clinical trial of the safety, pharmacokinetics and antiviral activity of pgt121 monoclonal antibody in HIV-uninfected and HIV-infected adults	Completed	…
N6LS	NA	NCT03538626	VRC 609: Phase I, open-label, dose-escalation study of the safety and pharmacokinetics of a human monoclonal antibody, VRC-HIVMAB091-00-AB (N6LS), administered intravenously or subcutaneously with or without recombinant human hyaluronidase PH20 (rHuPH20) to healthy adults	Recruiting	…
10E8	NA	NCT03875209	Phase 1 dose-escalation study of the safety, tolerability, pharmacokinetics, and antiviral activity of the bispecific antibody 10E8.4/iMab in HIV-1–infected and uninfected individuals	Recruiting	…
PG9	NA	NCT01937455	Phase 1, randomized, blinded, dose-escalation study of rAAV1-PG9DP recombinant AAV vector coding for PG9 antibody in healthy men	Completed	Priddy et al (2019) [28]
3BNC117	NA	NCT02018510	Phase 1, open-label, dose-escalation study of the safety, pharmacokinetics and antiretroviral activity of 3BNC117 monoclonal antibody in HIV-infected and HIV-uninfected volunteers	Completed	Caskey et al (2015) [16]
3BNC117	3BNC117 + 10-1074	NCT02824536	Phase 1 study of the safety and pharmacokinetics of the combination of 3BNC117 and 10-1074 in HIV-uninfected adults	Completed	Cohen et al (2018) [17]
3BNC117-LS-J	NA	NCT03254277	Phase 1 first-in-human study of the safety and pharmacokinetics of 3BNC117-LS in HIV-infected and HIV-uninfected individuals	Active, not recruiting	…
3BNC117-LS-J	3BNC117-LS-J + 10-1074-LS-J	NCT04173819	Safety and pharmacokinetics of the combination broadly neutralizing antibodies, 3BNC117-LS-J and 10-1074-LS-J, in healthy American and African adults	Recruiting	…
3BNC117-LS-J	3BNC117-LS-J + 10-1074-LS-J	NCT03554408	Phase 1, dose-escalation, first-in-human study of the safety and pharmacokinetics of the subcutaneous and intravenous administration of 10-1074-LS alone and in combination with 3BNC117-LS in HIV-infected and HIV-uninfected Individuals	Recruiting	…
10-1074	NA	NCT02511990	Phase 1, open label, dose-escalation study of the safety, pharmacokinetics and antiretroviral activity of 10-1074 monoclonal antibody in HIV-infected and HIV-uninfected individuals	Completed	Caskey et a (2017) [14]
2G12 (C2G12)	C2G12 + C2F5 + C4E10	ISRCTN64808733	Randomized double-blind phase 1 study to assess the pharmacokinetics of C2F5, C2G12, and C4E10 when administered together in a gel vehicle as a vaginal microbicide	Completed	Morris et al (2014) [10]
2G12 (P2G12)	NA	NCT01403792	Double-blind, placebo-controlled, randomized, dose-escalation phase I safety study of a single vaginal administration of P2G12 antibody in healthy female subjects	Completed	Ma et al (2015) [12]
2G12 (P2G12)	NA	NCT02923999	Phase I dose-escalation trial to evaluate safety and reactogenicity of single intravenous administration of P2G12	Not yet recruiting	…

Abbreviations: AAV, **adeno-associated viral vector;** HIV, **human immunodeficiency virus;** iMab, ibalizumab; **NA**, not applicable; PET, positron emission tomography.

A blank cell in the publication column indicates that there are no published data currently available.

### VRC01

Antibodies from the VRC01 class, which target the CD4-binding site of the HIV envelope, have been advanced in the clinical development pipeline [18]. Trials evaluating VRC01 administered both intravenously and subcutaneously at single or multiple dosing intervals have demonstrated safety with favorable pharmacokinetics [19, 20]. The first-in-human phase 1 dose-escalation study evaluating intravenous and subcutaneous administration of VRC01 in healthy adults was conducted at the National Institutes of Health. VRC01 was safe and well tolerated. The terminal half-life was 15 days over the 5-40-mg/kg intravenous dose range and 17 days with the 5-mg/kg subcutaneous dose. With repeated doses, higher trough values were seen across dosing groups [19].

Results from the HIV Vaccine Trials Network 104 trial also demonstrated the safety and tolerability of VRC01 administered intravenously at a dose range of 10–40 mg/kg monthly or bimonthly, or as a subcutaneous injection of 5 mg/kg every 2 weeks, in healthy, low-risk individuals. The terminal half-life was approximately 15 days. VRC01 demonstrated stable pharmacokinetics after multiple dosing and remained detectable at a concentration of ≥1.1 mg/mL in 60% of participants, 10 weeks after the last injection. In this trial, the investigators highlighted that more participants missed visits when allocated to receive more frequent injections, every 2 or 4 weeks, highlighting the need for acceptable dosing intervals [20].

### VRC01LS

VRC01LS, a modified version of VRC01 with an extended half-life, demonstrated safety with an expected terminal half-life that was 4-fold greater than that of the “parental” antibody, VRC01 [21]. Over the 5–40-mg/kg intravenous dose range, there was an elimination half-life of 71 days. The causal relationship between the extended half-life and reduced clearance demonstrated that less frequent, lower-dose, affordable administrations can be achieved with engineered variants. This trial also found that while the 5-mg/kg subcutaneous dose group had lower peak serum concentrations than the 5-mg/kg intravenous group, serum concentrations at week 4 were similar in both groups. The concentrations were >10 µg/mL, deemed protective, and persisted for up to 24 weeks in the subcutaneous treatment group.

### VRC07-523LS

A recent phase 1 trial assessed VRC07-523LS, an engineered variant of a clonal variant of VRC01 with wide coverage of clade B and C viruses [22]. The variant, called VRC07, was created by structure-based design and contains an insertion of 4 amino acids in the heavy-chain CDR3 and 16–amino acid mutations in the heavy-chain variable domain. Subsequently, VRC07 was extensively engineered to improve its biophysical properties and also modified to an LS version. VRC07-523LS was safe and well tolerated when administered both intravenously and subcutaneously in healthy adults. Although engineered antibody variants with structure-based designs can cause autoreactivity owing to small changes in amino acid sequences, this study demonstrated that VRC07-523LS was safe [23, 24]. Elimination half-lives of 38 and 33 days were observed for the intravenous and subcutaneous groups, respectively. Compared with VRC01 and VRC01LS, VRC07-523LS had favorable neutralization properties, but its serum half-life was unexpectedly shorter [21], possibly owing to additional modification of the engineered VRC07-523LS [25].

### 10E8VLS

A phase 1 study evaluating the subcutaneous administration of 10E8VLS alone or in combination with VRC07-523LS in healthy adults was suspended owing to safety concerns about local reactogenicity. Seven of 8 participants receiving 10E8VLS at 5 mg/kg subcutaneously experienced erythema and induration within 24 hours. A biopsy from 1 participant with induration demonstrated a nonspecific injection site reaction and panniculitis. Previous studies assessing other MPER bnAbs, 2F5, and 4E10, had demonstrated polyspecific autoantibodies reactive against the phospholipid cardiolipin similar to what is seen in autoimmune diseases [26]. Collectively, findings from these studies emphasize that investigators should proceed with caution when assessing future MPER bnAbs.

### 10E8 Bispecific and Trispecific Antibody

A trial investigating the bispecific antibody 10E8.4/iMab is currently recruiting participants; 10E8.4/iMab contains an antigen binding arm (ibalizumab [iMab]) that targets the CD4 receptor via the Fab ibalizumab, and a second antigen binding arm targets the MPER region Fab of 10E8. A phase I, first-in-human trial evaluating the trispecific antibody SAR441236 that incorporates VRC01, 10E8, and PGDM1400 with LS modification was initially paused owing to safety concerns from the 10E8VLS trial. However, the study proceeds in HIV-infected individuals, as there are fewer safety concerns with 10E8 as part of the trispecific antibody, possible because it contains only the binding region of the antibody (trial identifier NCT03705169)

### PGT121

Preliminary data from the phase 1 trial of PGT121 demonstrated safety with a favorable pharmacokinetic profile [27].The first part of this study included 20 HIV-negative participants who received 1 dose at 3, 10, and 30 mg/kg intravenously or 3 mg/kg subcutaneously. The half-life of PGT121 was 22 days, with variation by dose and route. Recently, a phase 1 trial evaluating an engineered variant of PGT121, elipovimab (formerly known as GS-9722), a first-in-class effector-enhanced bnAb, was presented (trial identifier GS-US-420-3902). Elipovimab, administered intravenously in 45 HIV-uninfected participants in single doses of 150, 500, or 1500 mg or multiple doses of 150, 500, or 1000 mg every other week, for 3 doses, was safe, with a half-life of 26 days [24]. A phase 1 trial of PGT121.414.LS is also currently underway (Table 1).

### Vectored Delivery bnAb Trials

Another milestone was achieved when a trial provided evidence to support further development of adeno-associated viral vectors (AAVs) for antibody gene delivery [28]. AAV1-PG9DP, a recombinant vector encoding the gene for the bnAb PG9, was safe and tolerable when administered intramuscularly in healthy men. However, anti-PG9 and anti-AAV1 antibodies as well as AAV1-specific T-cell responses were observed in 10 participants who received the higher dose. Although PG9 was not detected in serum by enzyme-linked immunosorbent assay, it was detected by HIV neutralization and by reverse-transcription polymerase chain reaction in muscle biopsy samples from 4 participants. Furthermore, although PG9 messenger RNA was detected at the injection site, there was low expression of the antibody in serum. The low circulating antibody levels and local antibody expression highlight the challenges with inducing protein expression. Another trial evaluating the safety of recombinant AAV vector expressing VRC07 (AAV8-VRC07) in HIV-1 infected adults virologically suppressed on ART is currently underway (trial identifier NCT03374202).

### Ongoing bnAb Combination Trials

Several antibody combination trials are also in the field (Table 1). A set of trials evaluating N6LS and 10-1074LS, alone and in combination with 3BNC117LS, are currently recruiting participants, as is a trial of CAP256V2LS administered alone and in combination with VRC07-523LS and PGT121. Further trials evaluating PGT121.414LS administered alone and in combination with VRC07-523LS are also planned. PGDM 1400 is already included in 2 recruited ongoing phase 1 studies in varying combinations with PGT121, VRC07-523LS 10-1074, and PGDM1400.

### Ongoing HIV Prevention Trials

Several bnAbs have demonstrated the ability to protect against HIV acquisition in animal models, but the exact concentration of bnAbs in the blood, plasma and tissue required to prevent HIV acquisition in humans is currently unknown [5, 29, 30]. SHIV challenge models used in NHP studies are not standardized, and the inhibitory concentrations are dependent on the sensitivity of the respective viruses against the bnAb used in the challenge models [5]. Although there have been significant advances in humanized mouse and NHP models, there is a lack of correlation between the bioavailability of biotherapeutics in animals and in humans [31–33].

Trials are based on NHP studies. The risks associated with undertaking trials based on a single NHP study was highlighted recently. Treatment with vedolizumab, a monoclonal antibody against α4β7 integrin, led to sustained suppression of SHIV plasma viremia. However, this finding could not be reproduced in humans with only a single trial participant showing prolonged suppression of plasma viremia after analytical treatment interruption. Parallel studies between animals and humans may be required to determine whether models are well representative of human pathogenesis. This concept of parallel human and NHP studies was used to define the optimal mosaic adenovirus serotype 26‐based HIV-1 vaccine regimen, which has now entered phase 2b and 3 studies in humans [34].

Results from the first test-of-concept studies conducted by the HIV Vaccine Trials Network and the HIV Prevention Trials Network in 2 parallel phase 2b prevention efficacy trials are expected in late 2020. These antibody-mediated prevention trials evaluated whether VRC01 prevents infection among 2700 HIV-uninfected men and transgender persons who have sex with men in United States, Peru, Brazil, and Switzerland and 1900 HIV-negative sexually active women from 7 sub-Saharan African countries. Participants were randomized 1:1:1 to receive either 10 mg/kg VRC01 intravenously, 30 mg/kg VRC01 intravenously, or placebo every 8 weeks for a total of 10 infusions [35]. These trials were designed to evaluate the overall prevention efficacy, to determine the correlates of protection and to identify threshold levels that are associated with high-level protection [36]. Although beyond the scope of this review, a host of prevention of mother-to-child transmission studies evaluating VRC01, VRC01LS, and VRC07-523LS are also underway [37–39].

## Discussion

### The Future of bnAb Research Against HIV

Data from several trials currently underway will contribute to advancing the antibody field [40]. Combination trials will help to clarify whether there is antagonism and/or synergy between antibodies, and whether broad coverage is required for neutralization and to prevent antibody resistance. The effects of engineered bnAbs, modified based on structure-based designs, will provide insight on whether the enhanced effects of such antibody modifications are safe. The use of engineered immunomodulatory proteins and vectors will provide insight into long-term antibody delivery and protection [41–43].

The development pathway for bnAbs as microbicides remains controversial. Microbicides could offer advantages, such as ease of application, targeted delivery, and cost-effectiveness [44]. Cheaper production was shown when 2G12 was expressed in a rice cell line that allowed crude seed extract to be used directly as a topical microbicide cocktail [45]. A phase I trial is currently evaluating the safety of HIV and Herpes Simplex Virus antibodies formulated in the MB66 vaginal ring, which may overcome the challenges of poor adherence seen in previous microbicide trials [46]. Furthermore, bnAbs that agglutinate sperm may be added to provide additional contraceptive protection [47].

Combining bnAbs with long-acting ART for a prevention indication should also be explored. Cabotegravir is being assessed as a sustained release preexposure prophylaxis agent. Currently, a phase 1 study is evaluating the safety and pharmacokinetics of cabotegravir with VRC07-523LS in virologically suppressed HIV-positive adults (trial identifier NCT03739996).

### The Ultimate Goal of bnAb Trials

Several bnAbs are currently in development with the hope of producing alternative HIV prevention strategies. Although they are promising, many unanswered questions and potential obstacles need to be addressed (Table 2). If the ultimate goal is to use results from these bnAb studies to design and develop an effective HIV vaccine, then this may be difficult to achieve. Studies have shown that bnAbs have unusual characteristics, such as insertions and/or deletions (indels), high levels of somatic hypermutation, and unusually long complementarity-determining regions H3, that pose challenges in vaccine design [48]. Furthermore, no vaccination strategies thus far have succeeded in eliciting effective bnAb responses [49]. Studies have predominately been conducted in high-income settings with limited phase 2b/3 data available yet. It is important to conduct bnAb trials in high-income as well as low- and middle-income countries, as data will collectively provide insight into the safety and efficacy of bnAbs when delivered to diverse populations.

**Table 2.** Potential Obstacles to the Use of Monoclonal Antibodies in Human Immunodeficiency Virus Prevention

Potential Obstacles	Responses
1. Can bnAbs effectively prevent HIV infection?	• The AMP trial will provide the first results on efficacy in humans. • Several more potent bnAbs are being investigated in clinical trials. • Combinations of bnAbs may be required to increase potency and breath. • In pregnant women, bnAbs may have possible advantages over ART. There may be possible protection against breast-milk transmission if the mother becomes infected soon after parturition and bnAbs are selectively transmitted with concentrations maintained in infants.
2. Are bnAbs safe when delivered to diverse populations at scale?	• Although bnAbs were found to be safe in several phase 1/2a studies, no phase 2b/3 data are yet available. • Limited safety studies have thus far been conducted in high-burden settings outside the United States. • Limited safety data are available on the delivery of engineered and vector-delivered antibodies, as well as antibodies delivered in combination. • An additional concern is the long tailing off of bnAb concentrations once discontinued. This can inadvertently lead to infections with variants that are less sensitive to ART.
3. Can bnAbs be delivered at sufficient concentrations to prevent HIV transmission and contain viremia?	• Intravenous administration may not be a practical route of administration, while the bioavailability of subcutaneous injections could pose a challenge. • Current limitations may be overcome with novel technology, such as longer-acting LS versions, vector-mediated delivery, or the use of excipients.
4. Can bnAb technology be implemented as an effective HIV prevention tool?	• Current manufacturing capacity must be able to provide sufficient product for large-scale cost-effective implementation. • The stability of bnAbs while in transit and on site must be maintained. • There must be feasibility and acceptability for the provider and end user with integration of other clinical services. • Laboratory testing must allow differentiation of bnAbs and HIV antibodies.

Abbreviations: AMP, antibody-mediated prevention; ART, antiretroviral therapy; bnAbs, broadly neutralizing antibodies; HIV, human immunodeficiency virus; LS, lysine-serine mutation.

If bnAbs are to be commercialized, the dose, route of administration, and bioavailability need to be considered, so that they are delivered at sufficient concentrations. The intravenous route is not always acceptable and is impractical at a population scale, whereas subcutaneous administration offers considerable client and resource benefits. The challenge with subcutaneous administration is the limited volume that can be administered. An interesting concept is the use of the enzyme hyaluronidase that temporarily hydrolyses hyaluronan and allows administration of increased volumes subcutaneously. Early hyaluronidase formulations were derived from animal products and associated with immunogenicity. However, the development of a purified recombinant soluble human formulation, rHuPH20 with ENHANZE drug delivery technology (Halozyme Therapeutics), has overcome these challenges. Several oncology trials have demonstrated favorable results, and trials evaluating CAP256V2LS and N6LS with the use of this technology will inform the HIV bnAb field.

If bnAb trials prove successful, a development and commercial manufacturing program that is cost-efficient at scale must be in place. The integration of a bnAb strategy into an existing care model that coincides with routine clinic visits, such as antenatal care and contraception provision, would be more practical and ensure feasibility and acceptability for providers and end users. This implementation should outweigh benefits of existing modalities, such as ART preexposure and postexposure prophylaxis. In 2019, ART was available at a cost of $75 per patient per year in low- and middle-income countries [50]. Although costs will decrease as bulk manufacture and production of bnAbs improve, cost analyses should be conducted, comparing short-term costs of bnAb provision with the costs of treatment and long-term care of an HIV-infected individual.

The potential of bnAbs in passive immunization and for vaccine development for HIV prevention is enormous. Unfortunately, the HIV clinical research field is currently globally disrupted by the coronavirus disease 2019 (COVID-19) pandemic. Researchers, in consultation with data safety monitoring boards and ethics committees, have had to make tough decisions to ensure the safety of participants and study staff, while protecting the integrity of the trials. Challenges have included the interrupted supply of investigational products and participant access to study sites during lockdowns, a global shortage of severe acute respiratory syndrome–covoronavirus 2 testing kits for participants and staff, potential confounding of adverse events and immunogenicity assessments due to COVID-19 infection, and the inability to efficiently monitor trial conduct. For example, a decision was made to terminate the antibody-mediated prevention trial slightly early, and not all participants received all their allocated bnAb doses.

Despite these challenges, many HIV prevention trials have continued follow-up of participants, supported by technology-based interventions, including telemedicine and virtual monitoring. Although there is a long way to go to end the HIV epidemic, bnAbs may play an important role to get us there.

## Notes

***Acknowledgment.*** All authors are investigators/protocol team members of the CAPRISA 012 Trial.

***Financial support.*** The Centre for the AIDS Programme of Research in South Africa (CAPRISA) 012 trial is supported by the European and Developing Countries Clinical Trials Partnership (grant RIA2017S) and the South African Medical Research Council’s Special Initiative on HIV Prevention Technology.

***Potential conflicts of interest.*** All authors: No reported conflicts. All authors have submitted the ICMJE Form for Disclosure of Potential Conflicts of Interest. Conflicts that the editors consider relevant to the content of the manuscript have been disclosed.
